# Supplementary material for: Cdhr1a and pcdh15b link photoreceptor outer segments with inner segment calyceal processes revealing a potential mechanism for cone-rod dystrophy
Source: bioRxiv. 2026 Jan 14:2024.07.26.605360. Preprint. [Version 2] doi: 10.1101/2024.07.26.605360 (PMC12871198; doi:10.1101/2024.07.26.605360)

bioRxiv preprint doi: https://doi.org/10.1101/2024.07.26.605360; this version posted January 14, 2026. The copyright holder for this preprint (which was not certified by peer review) is the author/funder, who has granted bioRxiv a license to display the preprint in perpetuity. It is made available under aCC-BY 4.0 International license.

Supplementary figure 1: Expression of USHR genes in the zebrafish retina.
Whole mount in situ hybridization of 5 dpf zebrafish larva for **A)** *cdhr1a*, **B)** *pcdh15b*, **C)** *pcdh23*, **D)** *ush1ga*, **E)** *harmonin*, **F)** *myo7aa*. Ventral images of the embryos are displayed. White arrows indicate expression in the outer nuclear layer of the retina.

Supplementary figure 2: Expression of *cdhr1a* and *cdhr1b*.
Whole mount in situ hybridization of 5 dpf zebrafish larva for **A-C)** *cdhr1a*, **D-F)** *cdhr1b* in WT, cdhr1a+/- or cdhr1a-/- individuals. Ventral images of the embryos are displayed. Yellow arrows indicate expression in the outer nuclear layer of the retina. *cdhr1a* retinal expression is lost in the homozygous mutant. *cdhr1b* expression is absent from the retina in wildtype and cdhr1a mutants.

Supplementary Table 1: WISH probe primer sets

cdhr1a	ATGAAGAATGCAAGGGAAATA	TAATACGACTCACTATAGGGTCCTTCTGGACTGATTTCCAATGC
pcdh15b	GGTGATGGATCCAGTTCAGTG	TAATACGACTCACTATAGGGTCACAGAACAGTGGACTGAGA
pcdh23	AGTGATTCAGATGATCGACGA	TAATACGACTCACTATAGGGTCATAACTCTGTGATCTCTAA
harmonin	CCTTAGTTTGGTGGGCACCAA	TAATACGACTCACTATAGGGTTAAAAGAATGTCACCTCATC
ush1ga	TTCTTGCCTTAATGTCTGTTT	TAATACGACTCACTATAGGGGCGCAGCTTTCACAAAACCAT
myo7aa	AAACAAGGACATTTTAACCAC	TAATACGACTCACTATAGGGGAGTCACATCGATCACTGGAC

bioRxiv preprint doi: https://doi.org/10.1101/2024.07.26.605360; this version posted January 14, 2026. The copyright holder for this preprint (which was not certified by peer review) is the author/funder, who has granted bioRxiv a license to display the preprint in perpetuity. It is made available under aCC-BY 4.0 International license.

Supplemental Figure 1

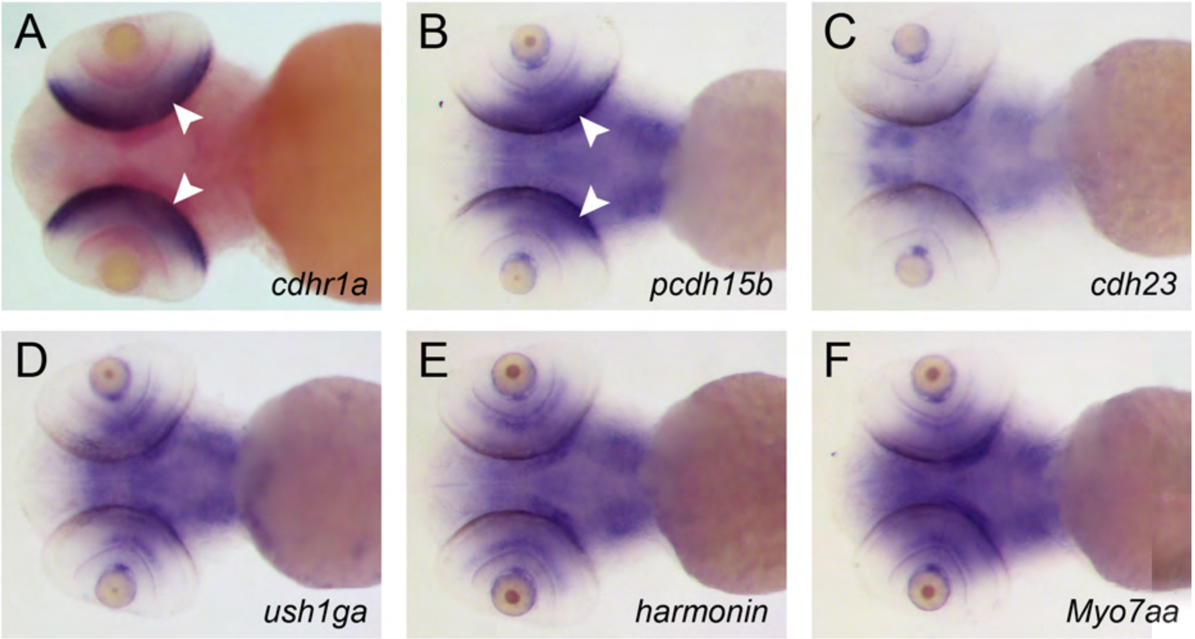

bioRxiv preprint doi: https://doi.org/10.1101/2024.07.26.605360; this version posted January 14, 2026. The copyright holder for this preprint (which was not certified by peer review) is the author/funder, who has granted bioRxiv a license to display the preprint in perpetuity. It is made available under aCC-BY 4.0 International license.

Supplementary figure 2

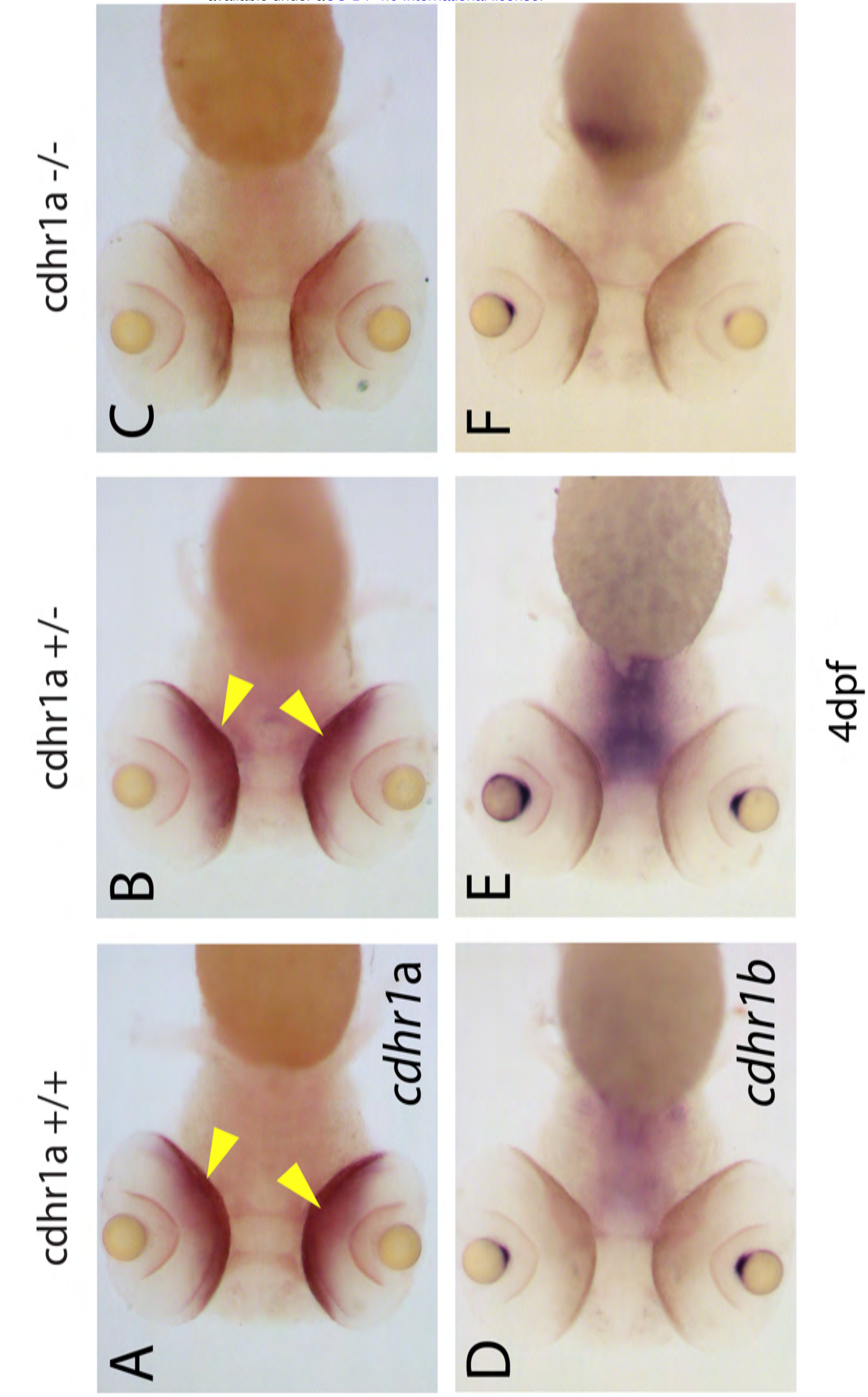

Supplement: Supplement 1 [file NIHPP2024.07.26.605360v2-supplement-1.pdf]
